# An All-Nanocrystal Biosensing System for In Vitro Detection of STAT3 Oligonucleotides

**DOI:** 10.3390/molecules22071085

**Published:** 2017-06-29

**Authors:** Wei Kong, Chau Fan Lam, Feng Wang

**Affiliations:** 1 Department of Physics and Materials Science, City University of Hong Kong, 83 Tat Chee Avenue, Hong Kong, China; weikong7-c@my.cityu.edu.hk (W.K.); chauflam4-c@my.cityu.edu.hk (C.F.L.); 2 City University of Hong Kong Shenzhen Research Institute, Shenzhen 518057, China

**Keywords:** lanthanide, nanocrystals, photoluminescence, energy migration, STAT3, DNA sensing

## Abstract

Lanthanide-doped nanocrystals have shown great promise in bio-detection due to their outstanding luminescent properties, including large Stokes shift and sharp emission bands. Herein, we describe an in vitro detection of STAT3 by using an all-nanocrystal biosensing system that takes advantage of inter-particle energy transfer between two types of lanthanide-doped nanocrystals. We investigate the effect of nanocrystal size on the sensing performance and find that smaller nanocrystals offer a lower detection limit and larger dynamic range. As STAT3 is identified as an oncogene aberrantly activated and expressed in malignant transformation and tumorigenesis, our study thus holds promise for cancer diagnosis and therapy.

## 1. Introduction

Lanthanide-doped luminescent nanocrystals that display highly tunable emissions have attracted numerous research interests in biological labelling [1,2], lasing [3,4], and solar energy conversion [5,6,7]. With transitions occurring within the 4f configuration that is shielded by the 5s^2^ and 5p^6^ shells, lanthanide-doped nanocrystals give rise to long excited-state lifetime, large Stokes and anti-Stokes shifts, sharp and narrow emission bands, and high photochemical stability. These attributes are particularly useful for bio-applications, as they can render high signal-to-noise ratio and minimize adverse biological effects [8,9,10,11]. Specifically, fluorescence resonance energy transfer (FRET)-based biosensing systems that use lanthanide-doped nanocrystals as energy donor in conjunction with organic dyes as an energy acceptor have been intensively studied [12,13,14,15,16]. However, these systems typically suffer from problems associated with organic dyes, such as low stability as well as broad absorption and emission bands that complicate interpretation of the spectra.

During the course of our investigation on energy migration in nanostructured materials, we discovered that energy transfer can be realized across two sets of nanoparticles comprising different lanthanide dopants [17]. The inter-particle energy transfer is dominated by the network of Gd^3+^ ions in the host lattice, which shortens the interaction distance between the donor and the acceptor. The findings have led to the development of a new class of biotin sensor consisting of lanthanide-doped nanoparticle probes [17]. Despite the encouraging achievements, further progress in utilizing such a biosensing system is constrained by the high detection limit and small dynamic range. In addition, the versatility of the biosensing system for detecting other molecules is largely unexplored.

**Figure 1:**
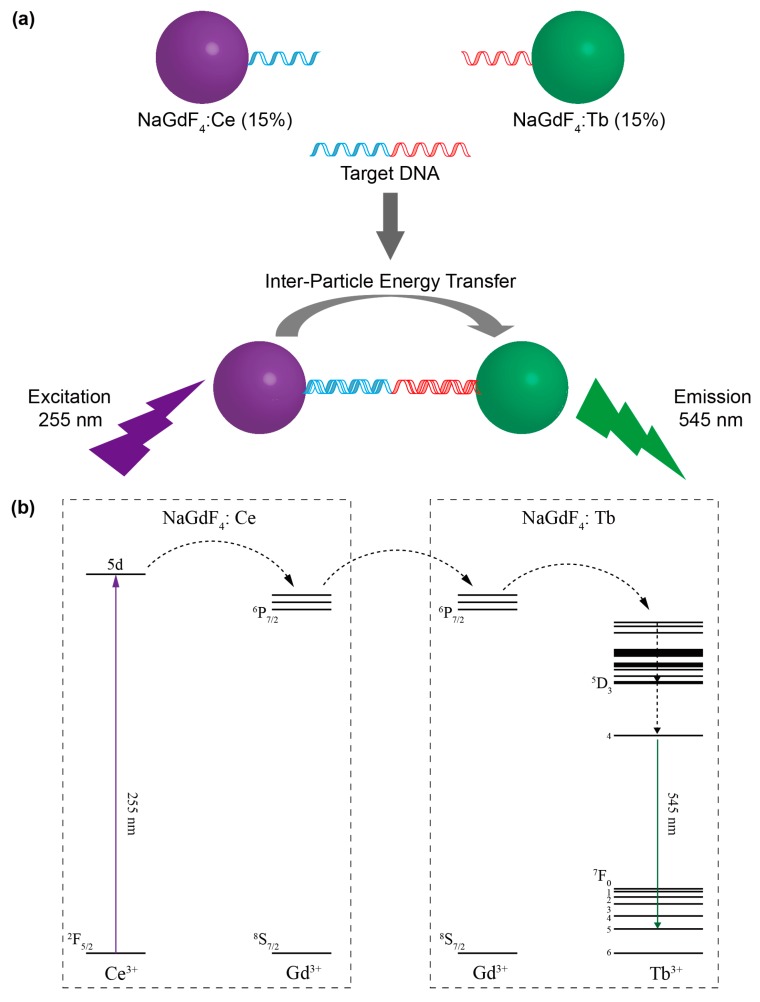
(**a**) Schematic illustration of the experimental design for DNA detection using oligonucleotide-functionalized nanoparticle probes; (**b**) Proposed energy transfer mechanism between NaGdF_4_:Ce and NaGdF_4_:Tb nanoparticles.

In this article, we describe a new class of DNA sensing system through the use of lanthanide-doped nanoparticles as both energy donor and acceptor. We also investigate the effects of nanoparticle size and concentration on the detection performance. The nanocrystals are functionalized with oligonucleotides that specifically bind to STAT3 (Figure 1). As an important member of the signal transducers and activators of transcription (STAT) protein family [18,19,20], STAT3 expression and signalling have been identified in tumours of the breast, ovary, pancreas, prostate, and melanoma. Based on recent research, the activation of STATs is tumour-specific and may represent a novel molecular target for the therapy and early diagnosis of tumours [21]. This study not only promotes the application of lanthanide-doped nanocrystals in biosensing, but also provides a promising means for the early diagnosis of tumours.

## 2. Results and Discussion

**Figure 2:**
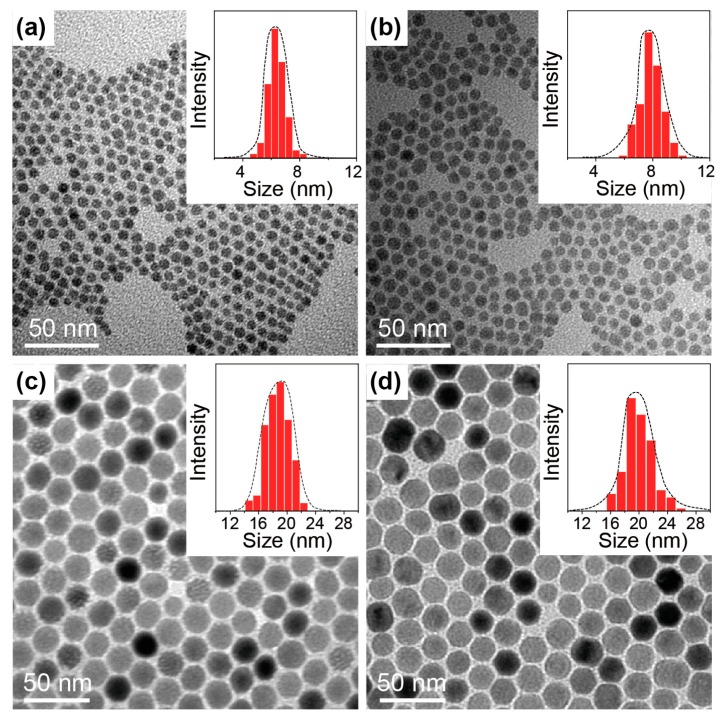
TEM image of the as-synthesized nanocrystals. (**a**,**b**) NaGdF_4_:Ce (15%) and NaGdF_4_:Tb (15%) nanocrystals synthesized in oleyamine/oleic acid/1-octadecene; (**c**,**d**) NaGdF_4_:Ce (15%) and NaGdF_4_:Tb (15%) nanocrystals synthesized in oleic acid/1-octadecene.

**Figure 3:**
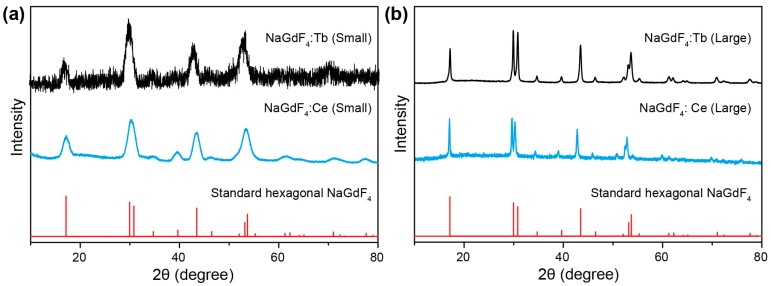
XRD pattern of the as-synthesized nanocrystals. (**a**) NaGdF_4_:Ce (15%) and NaGdF_4_:Tb (15%) nanocrystals synthesized in oleyamine/oleic acid/1-octadecene; (**b**) NaGdF_4_:Ce (15%) and NaGdF_4_:Tb (15%) nanocrystals synthesized in oleic acid/1-octadecene. The line spectra are literature data for hexagonal phase NaGdF_4_ crystals (Joint Committee on Powder Diffraction Standards file number 27-0699).

Lanthanide-doped NaGdF_4_ nanoparticles of varying sizes and dopant compositions were synthesized using a modified literature method by precipitating lanthanide ions with fluoride in organic solvents [22]. The small NaGdF_4_ nanoparticles (~8 nm) were synthesized in a ternary solvent mixture of oleyamine, oleic acid, and 1-octadecene, and the large NaGdF_4_ nanoparticles (~20 nm) were synthesized in a binary solvent mixture of oleic acid and 1-octadecene. The size control was dominated by oleyamine, which decreases the growth rate of hexagonal phase NaGdF_4_, according to a previous study [23]. As is shown in Figure 2, the as-synthesized nanocrystals all show uniform morphology and narrow size distribution. The average sizes of NaGdF_4_:Ce (15%) and NaGdF_4_:Tb (15%) synthesized in the presence of oleyamine were 6.5 nm and 8 nm, respectively, while the average sizes of NaGdF_4_:Ce (15%) and NaGdF_4_:Tb (15%) synthesized in the absence of oleyamine were 19 nm and 20 nm, respectively. The nanocrystals were further characterized by X-ray diffraction, and the results are shown in Figure 3. All samples exhibit diffraction patterns that can be easily indexed in accordance with hexagonal phase NaGdF_4_ crystals, suggesting high crystallinity and the single phase of the products.

**Figure 4:**
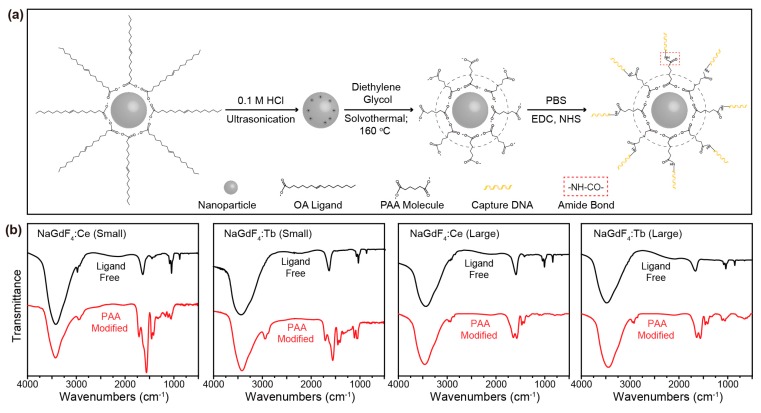
(**a**) Schematic illustration of the procedure of surface modification and DNA conjugation; (**b**) Fourier transform infrared (FTIR) spectra of ligand-free (black) and poly (acrylic acid) (PAA, red) modified nanocrystals with size of sub-10 nm (the first two) and over-10 nm (the last two). EDC: 1-ethyl-3-(3-dimethylaminopropyl) carbodiimide; NHS: *N*-hydroxysuccinimide; PBS: phosphate-buffered saline.

To functionalize the lanthanide-doped nanocrystals, oleyamine/oleate ligands capped on the as-synthesized nanocrystals were first exchanged with poly (acrylic acid) (PAA) molecules using a general strategy developed by our group [24]. In brief, this ligand exchange procedure comprises two steps; i.e., removal of origin hydrophobic ligands by ultra-sonication in hydrochloric acid solution and anchoring of PAA molecules onto the surface of the ligand-free nanocrystals through solvothermal treatment in diethylene glycol (Figure 4a). Fourier transform infrared (FTIR) spectra of the nanocrystals at different stages of the ligand exchange process are shown in Figure 4b. The spectra clearly show the successful attachment of PAA molecules as evidenced by the characteristic absorption bands at 2957 and 2925 cm^−1^ (asymmetric and symmetric stretching vibrations of CH_2_), 1638 cm^−1^ (asymmetric stretching vibration of CO_2_), and 1563 cm^−1^ (asymmetric stretching vibration of CO). The successful modification of PAA on the nanoparticle surface was further confirmed by zeta potential measurements (Table 1), which detect negative charges from the carboxylate groups.

After surface modification with PAA, capture oligonucleotides were conjugated with the nanoparticles (Figure 4a). In detail, the conjugation was accomplished by coupling the amino group of the capture oligonucleotides with the carboxylic group of PAA on the nanocrystal surface under the catalysis of 1-ethyl-3-(3-dimethylaminopropyl) carbodiimide (EDC) and *N*-hydroxysuccinimide (NHS) in a phosphate-buffered saline (PBS) buffer solution. The successful DNA conjugation was confirmed by a shift in surface charge as revealed by zeta potential measurements (Table 1).

**Figure 5:**
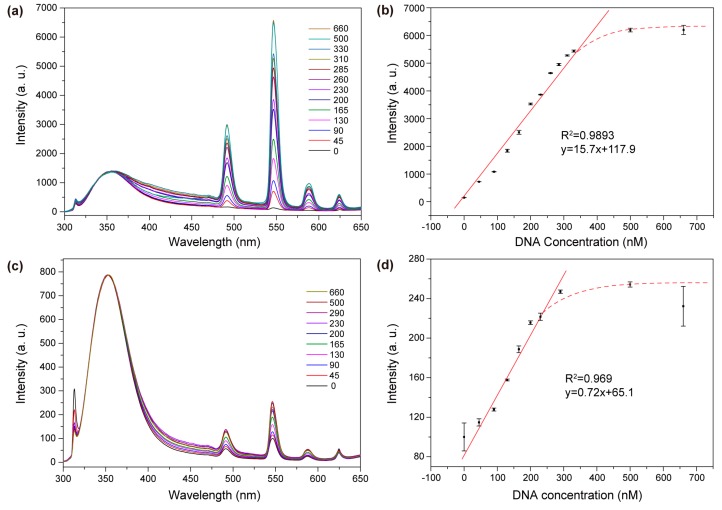
A comparison of the sensing performance by using small- and large-sized nanoparticle bioprobes (nanoparticle concentrations are fixed at 12.5 mM). (**a**) Emission spectra of the biosensing system composed of small-sized nanoparticle probes in the presence of varying concentrations of target DNA (nM); (**b**) Emission intensity of Tb^3+^ at 545 nm as a function of target DNA concentration from 0–660 nM. The straight line is a linear regression of the measured data in the DNA concentration range of 0–330 nM. The dashed curve marks the concentration range that deviates from the linear regression; (**c**) Emission spectra of the biosensing system composed of large-sized nanoparticle probes in the presence of varying concentrations of target DNA (nM) (**d**) Emission intensity of Tb^3+^ at 545 nm as a function of target DNA concentration from 0–660 nM. The straight line is linear regression of the measured data in a DNA concentration range of 0–230 nM. The dashed curve marks the concentration range that deviates from the linear regression. All the error bars shown represent the standard deviations from three sets of repeated measurements.

The performance of the biosensing system for STAT3 detection was evaluated in a standard in vitro assay at a nanoparticle concentration of 12.5 mM. Typically, capture DNA-conjugated NaGdF_4_:Ce and NaGdF_4_:Tb nanocrystals were first mixed in an aqueous solution. Prior to the addition of targeted DNA, the Tb^3+^ ions showed hardly any emission upon excitation into the Ce^3+^ ions at 254 nm. By addition of target DNA of increasing concentrations, the emission bands of Tb^3+^ ions at 490 nm (^5^D_4_→^7^F_6_), 545 nm (^5^D_4_→^7^F_5_), 586 nm (^5^D_4_→^7^F_4_), and 620 nm (^5^D_4_→^7^F_3_) ascended steadily due to the formation of DNA duplexes that bring the donor and acceptor nanocrystals into close proximity, enhancing the energy transfer processes (Figure 5a,c). At substantially high concentrations of target DNA, the Tb^3+^ emission is saturated due to the consumption of all free nanocrystals in the solution by forming DNA duplexes. By correlating the emission intensity of Tb^3+^ with the concentration of target DNA, we determined a dynamic range of 0–330 nM for the biosensing system using small-sized nanoparticle probes (Figure 5a,b). In addition, the detection limit of the assay—corresponding to an analyte concentration that provides a signal three times the standard deviation above the signal of the control experiments—was estimated to be 6.35 nM. In comparative experiments using large-sized nanoparticle probes, a dynamic range of 0–230 nM and a detection limit of 70.83 nM were determined, respectively (Figure 5c,d).

**Figure 6:**
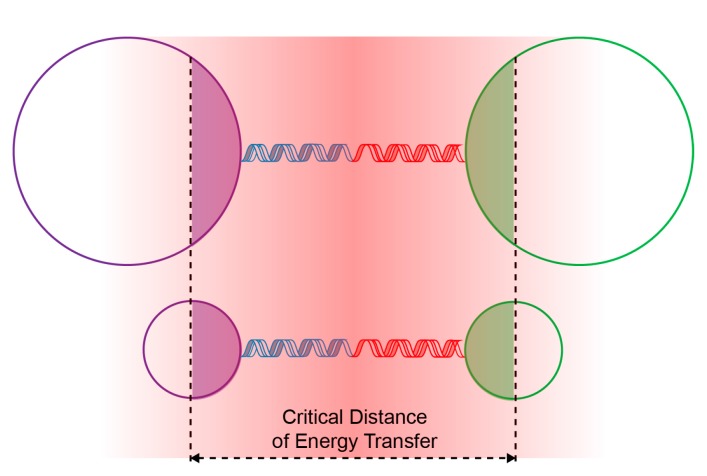
Schematic illustration comparing the inter-particle energy transfer for small- and large-sized nanoparticle probes.

The better performance (i.e., large dynamic range and low detection limit) achieved with the small-sized nanoparticle probes was ascribed to the higher surface-to-volume ratio, which results in a more effective inter-particle energy transfer and a high capacity to bind target DNA. As illustrated in Figure 6, a higher fraction of optical centres in the small-sized nanoparticles participate in the energy transfer process when compared to that in the large-sized nanoparticles. Therefore, the small-sized nanoparticle probes render a higher signal intensity than the large-sized nanoparticle probes at the same concentration of target DNA. Furthermore, the small-sized nanoparticles have a higher surface area than the large-sized nanoparticles at the same nanoparticle concentration, as confirmed by nitrogen physisorption measurements (125.4 versus 55.4 m^2^·g^−1^). Accordingly, the small-sized nanoparticle probes capture a larger amount of target DNA than the large-sized nanoparticle probes when all the free nanoparticles are consumed.

**Figure 7:**
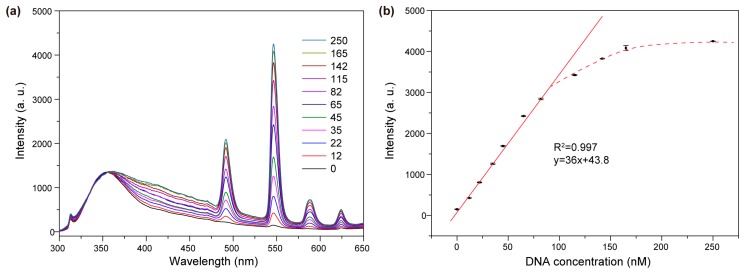
The performance of the biosensing system by using a low concentration of nanoparticle probes (6.25 mM). (**a**) Emission spectrum of the nanoparticle dispersion in the presence of varying concentrations of target DNA (nM); (**b**) Emission intensity of Tb^3+^ at 545 nm as a function of target DNA concentration from 0–250 nM. The straight line is a linear regression of the measured data in the DNA concentration range of 0–82 nM. The dashed curve marks the concentration range that deviates from the linear regression. All the error bars shown represent the standard deviations from three sets of repeated measurements.

In a further set of experiments, we investigated the effect of nanoparticle concentration on the sensing performance using small-sized nanoparticle probes. As shown in Figure 7, a reduction in the nanoparticle concentration to 6.25 mM resulted in a smaller dynamic range of 0–82 nM, which is ascribed to a reduced capacity of the nanoparticle probes to bind target DNA. Nevertheless, a lower detection limit of 1.08 nM was achieved with the diluted sensor system, which is comparable to what has been achieved with conventional sensing systems [25,26,27,28]. At a reduced nanoparticle concentration, a small amount of target DNAs bring a high fraction of nanoparticles into close proximity, leading to enhanced responses of the system.

**Figure 8:**
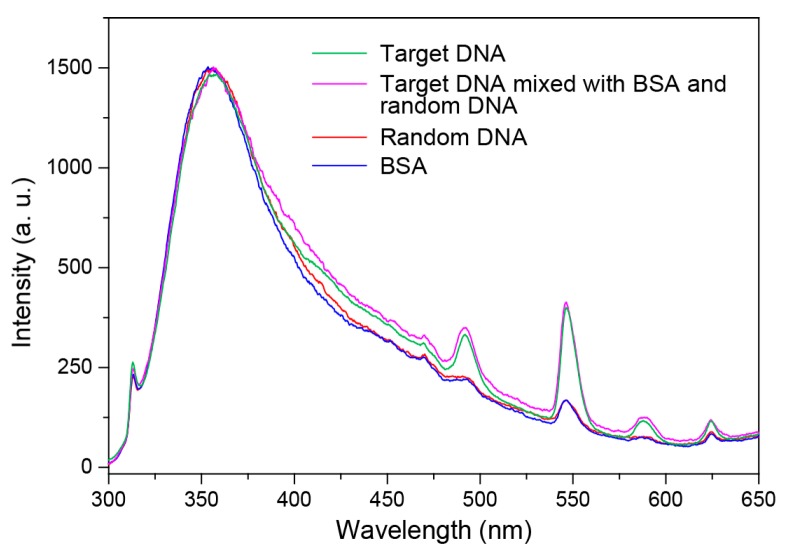
Emission spectra of the biosensing system in the presence of target DNA (green line), target DNA mixed with bovine serum albumin (BSA) and random DNA (purple), random DNA (red line), and BSA (blue line). The concentrations of nanoparticles and target DNAs are 6.25 mM and 12 nM, respectively. The sequences of employed random DNAs are 5′-CAAGGAGCTGGAAGGCTGGG-3′, 5′-ACATAAAGAAATCATGG-3′, and 5′-AGTTAAAGAAATCATGGAAGTAA-3′ (4 nM each).

We also conducted an interference study involving DNA strands of random sequences and bovine serum albumin (BSA). Emission spectra in Figure 8 reveal that the sensing response is highly specific to the target DNA, which demonstrates high resistance of the sensing system to interference.

## 3. Materials and Methods

### 3.1. Reagents

Gadolinium(III) acetate hydrate (99.9%), cerium(III) acetate hydrate (99.9%), terbium(III) acetate hydrate (99.9%), sodium hydroxide (NaOH, 98+%), ammonium fluoride (NH_4_F, 98+%), 1-octadecene (90%), oleic acid (90%), oleyamine (98+%), poly(acrylic acid) (PAA, MW ≈ 1800), phosphate-buffered saline (PBS), 1-ethyl-3-(3-dimethylaminopropyl) carbodiimide (98+%) and *N*-hydroxysuccinimide (98%) were purchased from Sigma-Aldrich (Shanghai, China). Targeted DNA oligonucleotide (5′-TCCCTGGACTTGATCTGCTG-3′) and amine-modified capture DNA oligonucleotides (5′-AGTCCAGGGA-NH_2_-3′ and 5′-NH_2_-CAGCAGATCA-3′) were purchased from Sangon Biotech (Shanghai, China) Co., Ltd. All reagents were used as received without further purifications.

### 3.2. Synthesis of Small NaGdF_4_:Ln (15%) (Ln = Ce and Tb) Nanocrystals

In a typical procedure, 2 mL water solution of Ln(CH_3_CO_2_)_3_ (0.2 M, Ln = Gd, Ce and Tb) was added to a 50 mL flask containing 2 mL of oleyamine, 3 mL of oleic acid, and 5 mL of 1-octadecene. The mixture was heated at 150 °C for 40 min to form the lanthanide–oleate complexes and then cooled down to 50 °C naturally. Thereafter, 5 mL of methanol solution containing NH_4_F (1.55 mmol) and NaOH (1 mmol) was added and the resultant solution was stirred for 30 min. After the methanol was evaporated, the solution was heated to 290 °C under argon for 1 h and then cooled down to room temperature. The resulting nanocrystals were precipitated by the addition of ethanol, collected by centrifugation at 6000 rpm for 5 min, washed with ethanol several times, and re-dispersed in 2 mL of cyclohexane.

### 3.3. Synthesis of Large NaGdF_4_:Ln (15%) (Ln = Ce and Tb) Nanocrystals

In a typical procedure, 2 mL water solution of Ln(CH_3_CO_2_)_3_ (0.2 M, Ln = Gd, Ce and Tb) was added to a 50 mL flask containing 4 mL of oleic acid and 6 mL of 1-octadecene. The mixture was heated at 150 °C for 40 min to form the lanthanide–oleate complexes and then cooled down to 50 °C naturally. Thereafter, 5 mL of methanol solution containing NH_4_F (1.55 mmol) and NaOH (1 mmol) was added and the resultant solution was stirred for 30 min. After the methanol was evaporated, the solution was heated to 290 °C under argon for 1 h and then cooled down to room temperature. The resulting nanocrystals were precipitated by the addition of ethanol, collected by centrifugation at 6000 rpm for 5 min, washed with ethanol several times, and re-dispersed in 2 mL of cyclohexane.

### 3.4. Synthesis of Ligand-Free Nanocrystals

One millilitre of a cyclohexane dispersion of the as-synthesized nanocrystals was precipitated by the addition of ethanol and re-dispersed in 2 mL of HCl solution (0.1 M in deionized water). The slurry was then sonicated at 45 °C for 1 h to remove the oleate ligands. After the reaction, the nanocrystals were collected via centrifugation at 14,000 rpm for 30 min, washed with deionized water twice, and re-dispersed in 1 mL deionized water.

### 3.5. Surface Modification of Nanocrystals with PAA

Typically, 50 mg of PAA molecules was dissolved in 9 mL of deionized water by adjusting the pH to 8 using NaOH solution (0.1 M in deionized water) under vigorous stirring at room temperature. Thereafter, 0.5 mL of ligand-free nanocrystals was added dropwise, followed by stirring for another 2 h. The water dispersion was then added to 10 mL of diethylene glycol (DEG) and the mixture was stirred at 105 °C for 1 h to remove water. Finally, the DEG dispersion was transferred to a 20 mL Teflon-lined autoclave and incubated at 160 °C for 2 h. The obtained nanocrystals were collected via centrifugation at 14,000 rpm for 30 min, washed with ethanol and deionized water several times, re-dispersed in 8 mL of deionized water, and stored in a fridge at 4 °C.

### 3.6. Synthesis of Capture DNA-Conjugated Nanocrystals

The bioconjugation of capture DNA with PAA-modified nanocrystals was conducted in the presence of 1-ethyl-3-(3-dimethylaminopropyl) carbodiimide (EDC) and *N*-hydroxysuccinimide (NHS). Typically, 1 mL of PAA capped-nanocrystals (12.5 mM) were mixed with 1 mmol NHS and 1.5 mmol EDC in 2.5 mL of PBS buffer solution (pH = 7). After stirring for 2 h, 1 mmol of capture DNA was added and the mixture was stirred for another 24 h. The capture DNA-conjugated nanocrystals were precipitated by centrifugation at 14,000 rpm and purified by washing with a mixture of water and ethanol (*v*/*v* = 1/1) twice. Finally, the DNA nanoconjugates were re-dispersed in 1 mL PBS buffer (pH = 7) for subsequent use in hybridization assays.

### 3.7. Detection of STAT3 by In Vitro Assay

Capture-DNA conjugated NaGdF_4_:Ce and NaGdF_4_:Tb nanocrystals (12.5 mM, 0.1 mL each) were mixed by stirring for 15 min. Then 0.1 mL of target-DNA of varying concentrations was added. After incubation for 1 h, the resultant mixture was subjected to spectroscopic examination in a quartz cuvette with 1 mm optical length under excitation at 255 nm.

### 3.8. Characterizations

Transmission electron microscopy (TEM) measurements were carried out on a Philips CM-20 transmission electron microscope (Philips, Amsterdam, Netherlands) operating at an acceleration voltage of 200 kV. Photoluminescence spectra were obtained from water dispersion of the nanocrystals (0.1 wt %) on a Hitachi F-4600 spectrophotometer (Hitachi, Tokyo, Japan). Zeta potential measurements were carried out on a Malvern Zetasizer Nano ZS (Malvern, Worcestershire, UK). Powder X-ray diffraction (XRD) pattern was collected on a Bruker AXS D2 Phaser X-ray diffractometer (Bruker, Karlsruhe, Germany) with Cu Kα1 radiation (λ = 0.154 nm). Fourier transform infrared (FTIR) spectra were obtained on a PE Spectrum 100 (PerkinElmer, Waltham, MA, USA). Nitrogen physisorption measurements were conducted at 77 K on automatic Nova 1200e (Quantachrome Instrument, Boynton Beach, FL, USA).

## 4. Conclusions

In conclusion, we have developed an all-nanocrystal biosensing system for the effective detection of a tumour-specific growth factor STAT3 DNA. The biosensing system takes advantage of inter-particle energy transfer from NaGdF_4_:Ce nanocrystals to NaGdF_4_:Tb nanocrystals that are enabled by the gadolinium host lattice. We find that small-sized nanoparticle probes provide a more effective inter-particle energy transfer and a high capacity to bind target DNA, thereby rendering a better detection performance. By using sub-10 nm NaGdF_4_ nanoparticle probes, we achieve a detection limit of 1.08 nM for STAT3 oligonucleotide. The current system can be readily adapted to detect other biomolecules through adjustment of the surface functionalization. Further improvement in the detection performance can be expected by using upconversion donors in combination with the optimization of nanoparticle size and concentration.

## Figures and Tables

**Table 1:** Zeta potential of the ligand-free, PAA-modified, and DNA-conjugated nanocrystals.

Sample	Zeta-Potential/mV		
	Ligand-Free	PAA-Modified	DNA-Conjugated
NaGdF_4_:Ce (Small)	47.7	−10.6	3.71
NaGdF_4_:Tb (Small)	52.5	−23.3	14.2
NaGdF_4_:Ce (Large)	46.8	−5.81	3.04
NaGdF_4_:Tb (Large)	48.2	−13.7	15.0
